# Risk factors for mortality in patients with *Stenotrophomonas maltophilia* bacteremia and clinical impact of quinolone–resistant strains

**DOI:** 10.1186/s12879-019-4394-4

**Published:** 2019-08-28

**Authors:** Eun Jin Kim, Yong Chan Kim, Jin Young Ahn, Su Jin Jeong, Nam Su Ku, Jun Yong Choi, Joon-Sup Yeom, Young Goo Song

**Affiliations:** 1 Department of infectious diseases, Ajou University School of Medicine, Suwon, Korea; 2 Department of Internal Medicine and AIDS Research Institute, Yonsei University College of Medicine, 50-1 Yonsei-ro, Seodaemun-gu, Seoul, 120-752 Republic of Korea

**Keywords:** Quinolone-resistant strains, *Stenotrophomonas maltophilia*, Bacteremia

## Abstract

**Background:**

*Stenotrophomonas maltophilia* is an important nosocomial pathogen. This pathogen has intrinsic or acquired resistance to a number of antibiotics classes. Furthermore, *Stenotrophomonas* infections have been associated with high mortality, mainly in immunocompromised patients. Accordingly, we conducted a retrospective cohort study on the clinical data, microbiological characteristics, and outcomes of patients with *S. maltophilia* (SM) bacteremia.

**Methods:**

A retrospective cohort study was conducted at two tertiary care referral hospitals in Seoul, South Korea. Data were collected between January 2006 and December 2015 from electric medical records*.* Our analysis aimed to identify the risk factors associated with crude mortality, as well as the predictive factors of quinolone-resistant strains in SM bacteremia patients.

**Results:**

A total of 126 bacteremia patients were enrolled in the study. The mortality rate was 65.1%. On multivariable analysis, hypoalbuminemia (odds ratio [OR], 5.090; 95% confidence interval [CI], 1.321–19.621; *P* = 0.018), hematologic malignancy (OR, 35.567; 95% CI, 2.517–502.515; *P* = 0.008) and quinolone-resistant strains (OR, 7.785; 95% CI, 1.278–47.432; *P* = 0.026) were independent risk factors for mortality. Alternatively, usage of an empirical regimen with quinolone (OR, 0.172; 95% CI, 0.034–0.875; *P* = 0.034) was an independent protective factor for mortality. The multivariable analysis of predictive factors revealed that high Charlson comorbidity index (OR, 1.190; 95% CI, 1.040–1.361; *P* = 0.011) and indwelling of a central venous catheter (CVC) (OR, 3.303; 95% CI, 1.194–9.139; *P* = 0.021) were independent predisposing factors associated with quinolone-resistant strains in SM bacteremia patients.

**Conclusions:**

Our findings suggest that a high Charlson comorbidity score and indwelling of a CVC were significantly independent predictors of quinolone-resistant strains in SM bacteremia patients*.* Therefore, we need to carefully consider the antibiotic use in SM bacteremia patients with these predictive factors.

**Electronic supplementary material:**

The online version of this article (10.1186/s12879-019-4394-4) contains supplementary material, which is available to authorized users.

## Background

*Stenotrophomonas maltophilia* is a non-fermentative, gram-negative bacillus that is closely related to the *Pseudomonas* species. *Bacterium bookeri*, now known as *S. maltophilia* (SM), was first isolated in 1943 and was subsequently classified as a member of the genus *Pseudomonas* in 1961. Thereafter, it was classified as a member of *Xanthomonas* genus in 1983, and finally it came to rest in the *Stenotrophomonas* genus in 1993 [1]. SM is a bacterium that can occur in almost any aquatic or humid environment [2], and is not considered to be a highly virulent pathogen. Over the last decade, SM has risen to prominence as an important nosocomial pathogen associated with significant case/fatality ratios in certain patient populations, particularly in individuals who are severely debilitated or immunosuppressed [3–5]. Pneumonia and bacteremia are the most common manifestations of SM infection [6]. However, treatment of SM bacteremia is challenging due to the resistance of SM to many broad-spectrum antimicrobial agents. Moreover, SM exhibits high-level intrinsic resistance to a variety of structurally unrelated antibiotics, including: beta-lactams, quinolones, aminoglycosides, tetracycline, disinfectants, and heavy metals [7, 8]. Furthermore, it can acquire resistance through the uptake of resistance genes located on integrons, transposons, and plasmids via horizontal gene transfer and mutations [9, 10]. Thus, choosing the optimal antibiotic for the treatment of SM bacteremia is very difficult. Trimethoprim-sulfamethoxazole (TMP-SMX) should be considered as the empirical choice for clinically suspected SM infections and as the treatment of choice for culture-proven infections by this agent [11, 12]. However, due to concerns regarding adverse events related to TMP-SMX treatment, levofloxacin has also been used as an alternative option [13, 14]. Fluoroquinolone and SMX monotherapies may be equally effective for the treatment of SM infections [15]. But, the overuse of quinolones worldwide has resulted in higher resistance rates in SM [16–18]. Therefore, we investigated the predictive factors of quinolone-resistant strains in SM bacteremia patients.

## Methods

### Study population and design

A retrospective cohort study was conducted at two tertiary care referral hospitals in Seoul, South Korea. Data were collected between January 2006 and December 2014 from digital medical records*.* Patients 18 years or older with 1 or more positive blood cultures of SM that met the Centers for Disease Control and Prevention (CDC) criteria for blood stream infection (BSI) [19], were eligible for inclusion. If a patient had multiple episodes of bacteremia, only the data from the first episode were included.

### Definitions

SM bacteremia was defined by the presence of a blood culture that yielded SM from one or more collected blood samples between January 2006 and December 2014. The source of bacteremia was determined clinically on the basis of the presence of an active site of infection as determined by chart review or isolation of the organism from other clinical specimens coincident with the episode of bacteremia. Polymicrobial bacteremia was defined by the isolation of an additional pathogen satisfying CDC criteria for BSI [19] within 24 h of index SM isolate. Healthcare-associated and community-acquired bacteremia were defined according to the CDC criteria for BSI [19]. Empiric antibiotic therapy was defined as the therapy initiated before the report of antibiotic susceptibility results, whereas definitive therapy was defined as the therapy given after the report of antibiotic susceptibility [20]. Chronic kidney disease was defined an estimated glomerular filtration rate (eGFR) (MDRD equation) of < 60 mL/min per 1.73m^2^ without renal replacement therapy. End stage renal disease was defined as an eGFR (MDRD equation) of < 15 mL/min per 1.73m^2^ with renal replacement therapy. Pulmonary disease was defined as chronic obstructive lung disease or asthma. Appropriate antimicrobial therapy was defined as the administration of at least one agent to which the index SM isolate was susceptible in vitro. Immunosuppressive therapy was defined as a daily ≥20 mg dose of a prednisolone-equivalent steroid, monoclonal antibodies, antimetabolite drugs, or T-cell inhibitors within 30 days prior to bacteremia onset. Neutropenia was defined as an absolute neutrophil count of < 500/mm^3^ at the onset of bacteremia. Thrombocytopenia was defined as a platelet count of less than 100,000/mL, and hypoalbuminemia was defined as an albumin count of less than 3 g/dL at the time of bacteremia. Mortality by SM bacteremia was defined as death within 60 days after the bacteremia isolation with no other apparent cause of death.

### Data collection

A trained examiner used a electric medical record to collect demographic data. Data elements included demographics, length of hospital stay before SM bacteremia (days), source of bacteremia, antibiotics-susceptibility of the isolated pathogen, antimicrobial therapy regimen, past medical history, comorbid conditions, surgery, prior chemotherapy/radiation therapy, or receipt of immunosuppressive medications (each 30 days). The data included appropriateness of antibacterial therapy, existence of indwelling medical devices before SM bacteremia during hospitalization, removal of previously present CVC, and length of stay in ICU and hospital (days). The degree of comorbidity was quantified using the Charlson comorbidity index [21] and the severity of illness was assessed by the Acute Physiology and Chronic Health Evaluation II (APACHE II) score. Laboratory data and outcomes were also recorded.

### In vitro antimicrobial susceptibility testing

All bacterial species were identified using conventional methods and/or the ATB 32GN system (bioMerieux, Marcy l’Etoile, France). Antimicrobial susceptibility tests were performed using the disk diffusion method or a VITEK-2 N131 card (bioMerieux, Hazelwood, MO, USA). The results were interpreted based on the Clinical and Laboratory Standards Institute (CLSI) guidelines [22]. SM isolates resistant to TMP-SMX were defined to have a minimal inhibitory concentration (MIC) ≥4/76 mg/mL to TMP-SMX. SM isolates which were resistant to levofloxacin (MIC ≥8 mg/mL) or those with intermediate resistance (MIC = 4 mg/mL) to levofloxacin were defined as quinolone-resistant strains.

### Statistical analysis

All statistical analyses were performed using SPSS for Windows (ver. 23.0, SPSS Inc., Chicago, IL, USA). The statistical analyses were performed to assess the factors associated with crude mortality and quinolone susceptibility. Continuous variables were expressed as mean ± standard deviation (SD), and categorical variables were expressed as a number (percentage). The Kolmogorov-Smirnov test was used to analyze the normality of the distribution of parameters. Data that did not show normal distributions were expressed as median and interquartile ranges (IQR). The Student’s t-test was used for continuous variables and the Chi-square test or Fisher’s exact test was used for categorical variables. Variables that did not show normal distributions were compared using the Mann-Whitney test or Kruskal-Wallis test. Univariate and multivariable logistic regression was used to evaluate the risk factors of mortality and the predictive factors of quinolone-resistant strains. Variables with *p*-values of less than 0.10 in univariate analyses were included in a multivariable logistic regression analysis to identify the risk factors associated with mortality and the predictive factors of quinolone-resistant strains. Results from the multivariate analysis are expressed as an odds ratio (OR) and 95% confidence interval (CI). All statistical tests were two-tailed and a *P* value of less than 0.05 was considered to be statistically significant.

## Results

### Baseline characteristics of SM bacteremia patients and comparison of clinical characteristics and outcomes

During the study period, 126 bacteremia patients were enrolled. The mean age of the patients was 61.22 ± 15.07 years and the 68.3% (86/126) of the patients were men. The mean length of stay in the hospital before the occurrence of SM bacteremia was 57.1 ± 22.5 days. The mean Charlson comorbidity index score was 6.5 ± 3.2 and the median APACHE II score was 13.0 (IQR, 9–19).

Bivariable analyses were performed to investigate the risk factors of mortality in SM bacteremia patients. Table 1 shows a comparison of survivors and non-survivors during the entire hospitalization period. Univariate analyses, showed that hematologic malignancy (*P* = 0.005), indwelling of hemodialysis catheter (*P* = 0.028), high APACHE II scores (*P* = 0.001), hypoalbuminemia (*P* = 0.003), thrombocytopenia (*P* = 0.004), and low hemoglobin concentration (*P* = 0.035) were associated with mortality. Also, the length of stay before bacteremia was longer in non-survivors (*P* = 0.038).

**Table 1:** Baseline characteristics of patients with *S. maltophilia* bacteremia

Factors	Survivors *N* = 44	Non-survivors *N* = 82	*p* value
Age, y, mean ± SD	71.0 (60.5–79)	74.5 (61.0–80.25)	0.310
Age ≥ 65 years, n(%)	24 (54.5)	34 (41.5)	0.160
Male, n (%)	33 (75.0)	53 (64.6)	0.233
BMI, kg/m^2^, median (IQR)	21.7 (19.4–25.0)	21.9 (19.0–24.4)	0.847
Comorbidities			
HTN, n (%)	15 (34.1)	32 (39.0)	0.585
DM, n (%)	16 (36.4)	23 (28.0)	0.336
Cardiovascular disease, n (%)	9 (20.5)	13 (15.9)	0.517
Chronic kidney diseasea, n (%)	4 (9.1)	4 (4.9)	0.449
End stage renal disease, n (%)	1 (2.3)	7 (8.5)	0.259
Chronic liver disease, n (%)	5 (11.4)	11 (13.4)	0.742
Pulmonary disease, n (%)	4 (9.1)	8 (9.8)	1.000
Solid tumor, n (%)	18 (40.9)	43 (52.4)	0.217
Hematologic malignancy, n (%)	1 (2.3)	17 (20.7)	0.005
Solid organ transplantation, n (%)	3 (6.8)	4 (4.9)	0.694
Charlson score, mean ± SD	5.7 ± 3.0	6.9 ± 3.2	0.049
Predisposing factors			
Chemotherapy, n (%)	4 (9.1)	10 (12.2)	0.769
Major surgery^a^, n (%)	15 (34.9)	29 (35.4)	0.957
ICU care, n (%)	29 (65.9)	54 (65.9)	0.995
Length of stay in hospital before bacteremia (days), median (IQR)	12.0 (3.5–24.5)	26.0 (14.0–56.0)	0.038
Central venous catheter, n (%)	31 (70.5)	59 (72.8)	0.777
Hemodialysis catheter, n (%)	2 (4.5)	15 (18.8)	0.028
Mechanical ventilator, n (%)	19 (43.2)	41 (50.6)	0.427
Foley catheter, n (%)	28 (63.6)	47 (58.0)	0.541
Clinical severity			
Shock, n (%)	10 (22.7)	33 (40.2)	0.075
APACHE II score, mean ± SD	11.0 ± 5.8	15.4 ± 6.9	< 0.001
Laboratory findings			
Neutropenia, n (%)	3 (6.8)	15 (18.3)	0.079
Hypoalbuminemia, n (%)	18 (6.8)	56 (18.3)	0.003
Thrombocytopenia, n (%)	9 (20.5)	38 (46.3)	0.004
Hemoglobin (g/L), mean ± SD	10.1 ± 1.6	9.4 ± 1.5	0.032
C-reactive protein (mg/L), median (IQR)	69.2 (32.2–129.5)	89.3 (58.5–148.0)	0.057
Estimated GFR (ml/min per 1.73m^2^), median (IQR)	85.0 (54.2–98.0)	80.0 (44.0–104.0)	0.675
Hospital stay, days	53.0 (20.5–78.0)	60.0 (35.0–97.0)	0.137
Length of stay in ICU, days	30 (11.5–50.0)	39 (23.0–67.0)	0.640

Note. *SD* Standard deviation, *BMI* Body mass index, *IQR* Interquartile range, *HTN* Hypertension, *DM* Diabetes mellitus, *ICU* Intensive care unit, *APACHE II* Acute Physiologic and Chronic Health Evaluation II score

^a^ Major surgery, any surgical procedure that involves anesthesia or respiratory assistance

Anatomic origin and microbiologic findings of SM bacteremia are presented in Table 2. Catheter-related infection was the most common primary source of bacteremia (49 cases, 38.9%), followed by intra-abdomen infection (38 case, 30.2%) and respiratory infection (35 case, 27.8%). The rate of CVC removal was 59.3% (54/91). In our study, 11.9% (15/126) of patients had SM strains resistant to TMP/SMX, and 31.2% (39/126) had strains resistant to quinolone. Only 21 patients (16.7%) were using an empirical regimen with quinolone and there were no cases using an empirical regimen with TMP/SMX. The number of patients under a definitive regimen with quinolone was 32% (40/126), and with TMP/SMX it was 24.8% (31/126).

**Table 2:** Anatomic origin and microbiologic findings of *S. maltophilia* bacteremia in study participants

Factors	Survivors *N* = 44	Non-survivors *N* = 82	*p* value
Infection source			
Pneumonia	12 (27.9)	23 (28.0)	0.987
Catheter-related infection	15 (34.1)	34 (42.0)	0.202
Intra-abdominal infection	16 (36.4)	22 (27.2)	0.285
Soft tissue infection	0 (0.0)	5 (6.2)	0.161
Urinary tract infection	1 (2.3)	0 (0.0)	0.352
Polymicrobial infection	18 (40.9)	36 (43.9)	0.746
Antibiotics susceptibility			
Quinolone resistance	5 (11.6)	34 (41.5)	0.001
TMP-SMX resistance	3 (6.8)	12 (14.6)	0.197
Resistant strain^a^	17 (38.6)	49 (59.8)	0.024
Treatment			
Empirical antibiotic use			
Cephalosporins	2 (4.5)	6 (7.4)	0.711
Carbapenems	13 (29.5)	42 (51.9)	0.016
Fluoroquinolones	13 (29.5)	8 (9.8)	0.004
BLBLIs	14 (31.8)	16 (19.5)	0.122
TMP-SMX	0 (0.0)	0 (0.0)	–
Definitive antibiotic use			
Carbapenems	2 (4.5)	16 (19.5)	0.022
Fluoroquinolones	21 (47.7)	19 (23.5)	0.005
BLBLIs	3 (6.8)	5 (6.2)	1.000
TMP-SMX	4 (9.1)	27 (33.3)	0.003
Inappropriate antimicrobial therapy^b^, yes	14 (31.8)	25 (30.9)	0.912

Note. *BLBLIs* Beta-lactam/beta-lactamase inhibitors, *TMP-SMX* Trimethoprim-sulfamethoxazole

The data were expressed as number (%) or median (interquartile range)

^a^Resistant strain, Quinolone or/and TMP-SMX resistance

^b^Appropriate antimicrobial therapy, the administration of at least one agent to which the index SM isolate was susceptible in vitro

### Mortality risk factors in SM bacteremia patients

On multivariable analysis, hypoalbuminemia (OR, 5.090; 95% CI, 1.321–19.621; *P* = 0.018), hematologic malignancy (OR, 35.567; 95% CI, 2.517–502.515; *P* = 0.008) and quinolone-resistant strains (OR, 7.785; 95% CI, 1.278–47.432; *P* = 0.026) were independent risk factors for mortality. Contrary, usage of an empirical regimen with quinolone (OR, 0.172; 95% CI, 0.034–0.875; *P* = 0.034) was independent protective factors for mortality (Table 3).

**Table 3:** Multivariate analysis for predictive factors for mortality in patients with *S. maltophilia* bacteremia

Factors	OR	95% CI	*P* value
Hematologic malignancy	35.57	2.52–502.52	0.008
Hypoalbuminemia	5.09	1.32–19.62	0.018
Quinolone resistance	7.79	1.28–47.43	0.026
Empirical antibiotic - quinolone use	0.17	0.03–0.88	0.034

Note. *OR* Odds ratio, *CI* Confidence interval

### Predicting factors for SM bacteremia with quinolone-resistant strains

Analyses of predictive factors for SM bacteremia with quinolone-resistant strains were performed (Table 4). One patient died immediately on the day of bacteremia and was excluded from this analysis due to death not related to antibiotic adequacy (*N* = 125 cases). On univariate analysis, high Charlson comorbidity index and long lengths of hospital stay before the onset of bacteremia were significant related factors (*P* = 0.030 and *P* = 0.015). Indwelling of CVC, ventilator, and Foley catheter were additional risk factors. Quinolone-resistant SM patients had a significantly higher mortality than the quinolone sensitive group (*P* = 0.001 and *P* = 0.013). Based on this multivariable analysis, the Charlson comorbidity index (OR, 1.190; 95% CI, 1.040–1.361; *P* = 0.011) and indwelling of a CVC (OR, 3.303; 95% CI, 1.194–9.139; *P* = 0.021) were identified as independent predisposing factors associated with quinolone-resistant strains in SM bacteremia patients.

**Table 4:** Comparisons of Clinical characteristics between quinolone-susceptible and quinolone-resistant groups

Factors	Susceptible group *N* = 86	Resistant group *N* = 39	*p* value
Age, years	63.0 (51.0–70.0)	66.0 (53.5–72.5)	0.215
Age ≥ 65 years, n(%)	37 (43.0)	20 (51.3)	0.390
Gender, male	57 (66.3)	29 (64.6)	0.233
Comorbidities			
HTN	30 (34.9)	16 (41.0)	0.509
DM	29 (33.7)	10 (25.6)	0.366
Cardiovascular disease	13 (15.1)	9 (23.1)	0.279
Chronic kidney disease	6 (7.0)	2 (5.1)	1.000
End stage renal disease	4 (4.7)	4 (10.3)	0.255
Chronic liver disease	12 (14.0)	4 (10.3)	0.774
Pulmonary disease	8 (9.3)	4 (10.3)	1.000
Solid tumor	42 (48.8)	19 (48.7)	0.990
Hematologic malignancy	12 (14.0)	6 (15.4)	0.833
Solid organ transplantation	5 (5.8)	2 (5.1)	1.000
Charlson score	6.1 ± 3.1	7.4 ± 3.2	0.030
Predisposing factors			
Chemotherapy	11 (12.8)	3 (7.7)	0.546
Major surgery^a^	28 (32.9)	16 (35.4)	0.382
ICU care	54 (62.8)	29 (74.4)	0.205
Hospital stay before bacteremia, days	17.0 (8.0–34.0)	30.0 (13.5–57.5)	0.015
Central venous catheter	57 (66.3)	32 (84.2)	0.019
Hemodialysis catheter	9 (10.5)	8 (21.6)	0.100
Mechanical ventilator	36 (41.9)	24 (63.2)	0.029
Foley catheter	47 (54.7)	28 (73.7)	0.046
Clinical findings			
Shock	23 (26.7)	20 (51.3)	0.007
APACHE II score	13.0 (8.0; 18.0)	14.0 (11.0; 21.5)	0.115
Laboratory findings			
Neutropenia	14 (16.3)	4 (10.3)	0.374
Hypoalbuminemia	52 (60.5)	21 (53.8)	0.487
Thrombocytopenia	28 (32.6)	19 (48.7)	0.084
Hemoglobin, g/L	9.8 ± 1.7	9.4 ± 1.3	0.296
C-reactive protein, mg/L	86.8 (41.5–142.5)	79.3 (44.5–153.0)	0.914
Estimated GFR, ml/min per 1.73m^2^	87.0 (51.0–101.0)	72.0 (45.4–106.0)	0.592
Outcomes			
Inappropriate antimicrobial therapy	23 (26.7)	16 (42.1)	0.089
Hospital stay, days	50.0 (26.0–78.0)	68.0 (35.0–150.0)	0.054
Mortality	48 (55.8)	34 (87.2)	0.001

Note. *HTN* Hypertension, *DM* Diabetes mellitus, *ICU* Intensive care unit, *APACHE II* Acute Physiologic and Chronic Health Evaluation II score

The data were expressed as mean ± standard deviation, number (%), or median (interquartile range)

^a^ Major surgery, any surgical procedure that involves anesthesia or respiratory assistance

## Discussion

Among non-fermenting gram negative bacilli, SM has been reported to be the third most commonly isolated pathogen after *Pseudomonas aeruginosa* and *Acinetobacter baumannii* [23]. Furthermore, SM bacteremia is associated with high mortality rates, the crude mortality estimates range from 21 to 69% [23]. Previous studies have reported that risk factors associated with mortality for SM bacteremia include indwelling of CVC in intensive care units, immunocompromising conditions, exposure to broad-spectrum antibiotic therapy, and long hospital stays [3, 9, 23]. In our study, the mortality rate of 65.1% was similar to the rate reported in previous studies. Bivariable analyses to investigate the risk factors of mortality in SM bacteremia, identified hematologic malignancy and hypoalbuminemia as independent risk factors. In addition, our results indicate that quinolone resistance has an impact on mortality, and it has also been shown that the use of empirical quinolone can reduce mortality after adjusting the analysis. There were also statistical differences in crude (87.2% vs 55.8%, *P* = 0.001) and attributable mortality (61.5% vs 30.6%, *P* = 0.001) between quinolone-resistant and sensitive groups. Consequently, we analyzed the risk factors associated with quinolone resistance in SM bacteremia. To our knowledge, there are no reports investigating the risk factors for quinolone-resistant SM bacteremia. There have been studies of risk factors related to the acquisition of resistance to levofloxacin in all clinical specimens and to quinolone susceptibility using only respiratory tract specimens with SM [17, 24]. In respiratory tract specimens, a relative factor for resistance to quinolone was the previous use of piperacillin/tazobactam, and risk factors to levofloxacin resistance were exposure to levofloxacin for more than 3 weeks and co-infection/co-colonization with *Klebsiella pneumoniae* resistant to levofloxacin.

In this report, a high Charlson comorbidity index and indwelling of CVC were independent risk factors associated with resistance to quinolone in SM bacteremia. The Charlson comorbidity index is a widely used comorbidity index [21]. A high CCI value means that many coexistent diseases may directly or indirectly affect the choice of antibiotics used and the outcome [25]. Especially, the presence of many coexistent diseases suggests that the previous use of antibiotics, or a prolonged history of hospital admissions, provides opportunities for coexistent diseases to develop. Furthermore, a high CCI value is associated with a greater risk to acquire common antibiotic resistance. This may be due to previous antibiotic uptake or hospital stays, however, this analysis was not part of our study and further studies are needed.

Indwelling of CVC has already been shown to be significantly associated with mortality and bacteremia in previous literature [9]. In particular, removal of CVC has been associated with reduced mortality [26–28]. However, removal of CVC did not influence mortality and quinolone resistance in this report. It is estimated that the rate of patients who adequately removed CVC was only 40%, and therefore did not significantly affect the outcome. SM has the ability to adhere on prosthetic devices such as CVC and form a biofilm. Biofilms can increase antibiotic resistance [11]. Therefore, indwelling of CVC is a risk factor to biofilm formation and, accordingly, to quinolone-resistance. Therefore, it is necessary to actively encourage the removal of CVC.

Previous literature has proven the relationship between inappropriate antimicrobial therapy and mortality in SM infections [23, 27]. Although not relevant to this study, usage of empirical quinolone had a significantly reduced mortality risk. Empirical antibiotic regimens are determined by the severity of patients. Therefore, patients that underwent an empirical quinolone regimen were more likely to have low SM severity and lower mortality. Overall, the periods of empirical antibiotic use were short and there were no patients using TMP / SMX empirically, so whether the empirical use of quinolone actually had a significant impact needs to be analyzed further. Also, SM infections may not be an independent contributor to mortality increase, therefore inappropriate therapy should not have major effects on the outcome of patients [29]. The rate of polymicrobial infections in SM bacteremia was high in the previous literature [23] and in this study as well (42.8%). Thus, high polymicrobial infections may be a confounding factor for appropriate antibiotic use, and this may have affected the mortality risk-factor analysis. Therefore, subgroup analysis was performed in patients with polymicrobial infection. However, there was no significant difference between the two groups regarding mortality (Additional file 1: Table S1).

Significantly, the use of empirical or definitive treatment of carbapenem was higher in the mortality group, although it was not significant in the multivariate analysis. This suggests that the possibility of breakthrough infections by SM in patients being treated with carbapenem is due to intrinsically resistant carbapenem and the selection pressure of SM should be considered [29, 30]. Only 24.8% of patients received TMP/SMX. A high proportion of ICU care and polymicrobial infections may have influenced antibiotic selection. Nevertheless, careful use of carbapenem is necessary, and the possibility for breakthrough infections should be considered.

Our study had several limitations. First, the retrospective design of this study was a major limitation. Second, the history of previous antibiotic use and previous hospital admissions were not investigated. This is especially important, since these are factors related to the acquisition of antibiotic resistance. Third, we could not investigate the susceptibility of other antibiotics except quinolone and TMP/SMX, and our quinolone analysis was limited to levofloxacin only.

## Conclusion

Quinolone-resistant SM isolates have been emerging and spreading in Korean hospitals, and current therapeutic options are limited for SM bacteremia. Our results suggest that a high Charlson comorbidity index and indwelling of CVC were significant independent predictors of SM bacteremia patients with quinolone-resistant strains*.* Therefore, we need to carefully consider antibiotics use in patients with SM bacteremia who have these predictive factors.

## Additional file

Additional file 1: **Table S1.** Clinical characteristics of polymicrobial infection groups. (DOCX 22 kb)

## Data Availability

The datasets used during the current study are available from the corresponding author on reasonable request.

## Abbreviations

APACHE II: Acute Physiology and Chronic Health Evaluation II
BSI: Blood stream infection
CDC: Centers for Disease Control and Prevention
CI: Confidence interval
CVC: Central venous catheter
eGFR: Estimated glomerular filtration rate
IQR: Interquartile range
MIC: Minimal inhibitory concentration
OR: Odds ratio
SD: Standard deviation
SM: *S. maltophilia*
TMP-SMX: Trimethoprim-sulfamethoxazole
