# Supplementary material for: Risk factors for mortality in patients with Stenotrophomonas maltophilia bacteremia and clinical impact of quinolone–resistant strains
Source: BMC Infect Dis. 2019 Aug 28;19:754. doi: 10.1186/s12879-019-4394-4 (PMC6714101; doi:10.1186/s12879-019-4394-4)
Supplement: Supplementary file 1 — Table S1. Clinical characteristics of polymicrobial infection groups. (DOCX 22 kb) [file 12879_2019_4394_MOESM1_ESM.docx]

**Additional file 1: Table S1. Clinical characteristics of polymicrobial infection groups**

| **Factors** | **Survivors**  **N = 18** | **Non-survivors**  **N = 36** | ***p* value** |
| --- | --- | --- | --- |
| Age, years | 58.1 ± 4.2 | 62.3 ± 2.5 | 0.359 |
| Gender, male | 14 (77.8) | 23 (65.7) | 0.530 |
| Infection source |  |  |  |
| Pneumonia | 4 (22.2) | 10 (28.6) | 0.701 |
| Catheter-related infection | 9 (50.0) | 15 (42.9) | 0.571 |
| Intra-abdominal infection | 5 (27.8) | 9 (26.5) | 1.000 |
| Soft tissue infection | 0 (0.0) | 2 (5.9) | 0.538 |
| Co-pathogen |  |  |  |
| *Pseudomonas* spp. | 2 (11.1) | 9 (26.2) | 0.291 |
| *Enterobacter* spp. | 0 (0.0) | 1 (2.9) | 1.000 |
| *Klebsiella* spp. | 1 (5.6) | 3 (8.6) | 1.000 |
| *Citrobacter* spp. | 1 (5.6) | 0 (0.0) | 0.346 |
| *Acinetobacter baumannii* | 4 (22.2) | 2 (5.9) | 0.166 |
| *Enterococcus* spp. | 2 (11.1) | 8 (23.5) | 0.462 |
| *Staphylococcus aureus* | 1 (5.6) | 2 (5.9) | 1.000 |
| *Coagulase-negative Staphylococcus* | 5 (27.8) | 6 (17.6) | 0.482 |
| *Streptococcus* spp. | 1 (5.6) | 1 (2.9) | 0.543 |
| *Candida* spp. | 1 (5.6) | 4 (11.4) | 0.648 |
| Clinical findings |  |  |  |
| Shock | 8 (44.4) | 18 (51.4) | 0.773 |
| Quinolone resistance | 3 (16.7) | 16 (45.7) | 0.068 |
| Remove central venous catheter, n (%) | 11 (61.1) | 16 (45.7) | 0.387 |
| Neutropenia | 1 (5.6) | 5 (14.3) | 0.418 |
| Hypoalbuminemia | 13 (72.2) | 25 (71.4) | 0.952 |
| Thrombocytopenia | 5 (27.8) | 19 (54.3) | 0.085 |
| Empirical fluoroquinolone use | 6 (33.7) | 3 (8.6) | 0.048 |
| Inappropriate antimicrobial therapy | 5 (27.8) | 15 (44.1) | 0.370 |

Data are expressed as mean ± standard deviation, number (%)
